# Higher-Than-Usual Target International Normalized Ratio (INR) Range Required With On-X Aortic Valve Secondary to Recurrent Thromboembolic Strokes: A Case Report

**DOI:** 10.7759/cureus.68546

**Published:** 2024-09-03

**Authors:** Sidra Hashmi, Aisha Rehman, Neelofar Iqbal, Ahsan Ali, Anoshia Raza

**Affiliations:** 1 Internal Medicine, Duke University Health System, Durham, USA; 2 Medicine, Doctors Hospital at Renaissance, McAllen, USA; 3 Medicine, Dow Medical College, Dow University of Health Sciences, Karachi, PAK; 4 Neurology, Doctors Hospital at Renaissance, McAllen, USA; 5 Cardiology, St. Mary's General Hospital, Passaic, USA

**Keywords:** lack of aspirin in on-x aortic valve, stroke despite increased inr, higher than usual target inr, recurrent ischemic stroke, recurrent thromboembolic stroke, on-x aortic valve

## Abstract

Although the On-X aortic valve (AO) is considered less thrombogenic compared to its counterparts, we present a case where recurrent thromboembolic ischemic stroke occurred, first with a *slightly* sub-therapeutic, then even with an elevated International Normalized Ratio (INR).

A 36-year-old male, the background of On-X AO replacement but no other risk factors, developed thromboembolic stroke twice while on Warfarin, first with INR 1.4, second with INR 2.4. Despite extensive investigation, other than elevated levels of low-density lipoproteins, no other treatable cause was found with the latter episode. The INR range was increased to 2.5-3.5, and aspirin and statin were added.

The occurrence of thromboembolic stroke with an On-X AO despite maintaining an INR of 2.4, presents a dilemma for future prevention. The American Heart Association (AHA) and the American College of Cardiology (ACC) guidelines for thromboembolism prevention in case of an On-X AO recommend an INR range of 1.5-2 as being effective when warfarin is used along with aspirin.

The take-home message is that the recommendation of an INR range of 1.5-2 with an On-X AO should be approached with caution; aspirin should be strongly considered regardless of the presence of thromboembolic risk factors. Patients developing thromboembolism have a high risk of recurrence. Therefore, a higher INR, along with the addition of aspirin and statin should be considered. Studies are needed to establish guidelines for a reliable INR range in these scenarios.

## Introduction

Mechanical heart valves are a known risk factor for thromboembolic phenomena. A newer generation valve, the bi-leaflet On-X aortic valve (AV), has been reported to improve valve hemodynamics and reduce thrombogenicity. It is composed of pure pyrolytic carbon without silicon and is designed to reduce turbulence and enhance blood flow across the valve [1]. Multiple studies have been published in favor of its efficacy and improved hemodynamics, leading to the acceptance of reduced anticoagulation levels compared to conventional mechanical heart valves. The results of the Prospective Randomized On-X Anticoagulation Clinical Trial (PROACT) showed that in the case of On-X AV replacement, the International Normalized Ratio (INR) can be safely maintained between 1.5 and 2.0, with no significant increase in thromboembolism risk when warfarin is used with aspirin [2].

We report a case of a young male with a past surgical history of On-X AV replacement, where recurrent thromboembolic ischemic stroke occurred, first with a slightly sub-therapeutic and then even with an elevated INR.

## Case presentation

A 36-year-old, right-handed male presented to the emergency room in January 2023 with a three-hour history of sudden-onset expressive aphasia. A physical exam showed expressive aphasia with no other neurologic issues.

The patient had a past history of AV insufficiency secondary to a congenital ventricular septal defect (VSD), which led to left ventricular dilation and aortic root dilation, as well as a sinus of Valsalva aneurysm and a patent foramen ovale. He had undergone surgical repair of the VSD and sinus of Valsalva aneurysm, right ventricular outflow tract bundle resection, and On-X AV replacement (25 mm Regent mechanical valve) in August 2021. Warfarin was initiated three months post-surgery due to the mechanical heart valve, with the target INR range set at 1.5-2, as recommended by the American Heart Association (AHA) and American College of Cardiology (ACC) guidelines. There was no history of blood disorders, prior thromboembolic episodes, or other thromboembolic risk factors, such as older-generation valves, atrial fibrillation, previous thromboembolism, a hypercoagulable state, or left ventricular systolic dysfunction [3], that would place the patient in the *high-risk* category.

**Figure 1:**
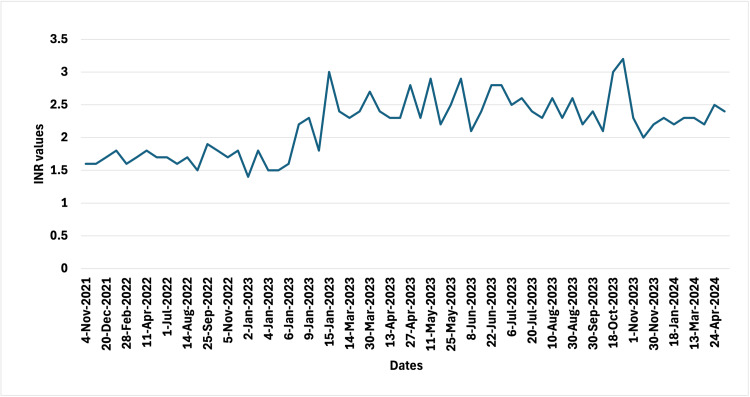
Graphical representation of the patient's INR values. INR, International Normalized Ratio

INR was generally maintained within range but difficulty in obtaining sufficient blood samples at home caused challenges in monitoring INR over the last three weeks. INR on presentation was 1.4 (slightly subtherapeutic). The patient confirmed consistent and minimal intake of food rich in vitamin K, i.e., green vegetables and moderate protein intake. Figure 1, a graph of INR values, shows that the least INR was 1.4, but readings otherwise stayed within range.

**Figure 2:**
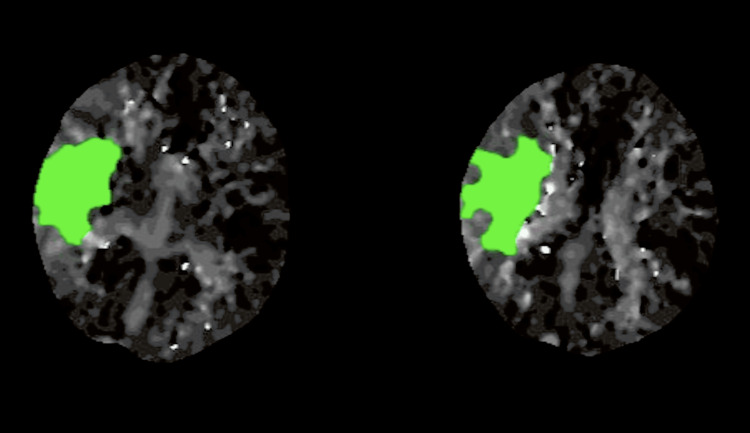
CT perfusion imaging of the brain. CT, computed tomography

The possible differentials could be an ischemic or hemorrhagic stroke. Computed tomography (CT) of the brain ruled out hemorrhage. CT angiography revealed an occlusion in the right middle cerebral artery. A CT perfusion scan further confirmed an ischemic penumbra in the corresponding territory, with a small core infarct (Figure 2).

Intravenous tissue plasminogen activator (tPA) was given, but symptoms persisted. Endovascular thrombectomy was subsequently done through which the clot was retrieved. Symptoms improved post-procedure, and the patient returned to baseline. 

A transthoracic echocardiogram was performed but could not clearly visualize the mechanical AV. However, no regurgitation or thrombus was visible, though the sensitivity of the test was considered very low. The echocardiogram also revealed a patent foramen ovale with mild left-to-right shunting (a known pathology in the patient) and showed that the VSD repair had no residual flow.

The target INR was increased from 1.5-2 to 2-2.5, and oral aspirin was added. However, aspirin was later discontinued due to the absence of other stroke risk factors, such as advanced age, obesity, hypertension, diabetes, and a history of smoking, as well as because the cause of the thrombosis was identified as a sub-therapeutic INR.

A year later, in April 2024, the patient presented to the emergency room with acute onset dizziness and mild, short-lived blurry vision. The history confirmed consistent maintenance of INR within the suggested therapeutic range of 2-2.5, with the current INR being 2.4 (within range). A CT angiogram of the head revealed a short-segment occlusion of the right posterior cerebral artery, P2-segment, with distal reconstitution via collaterals.

**Figure 3:**
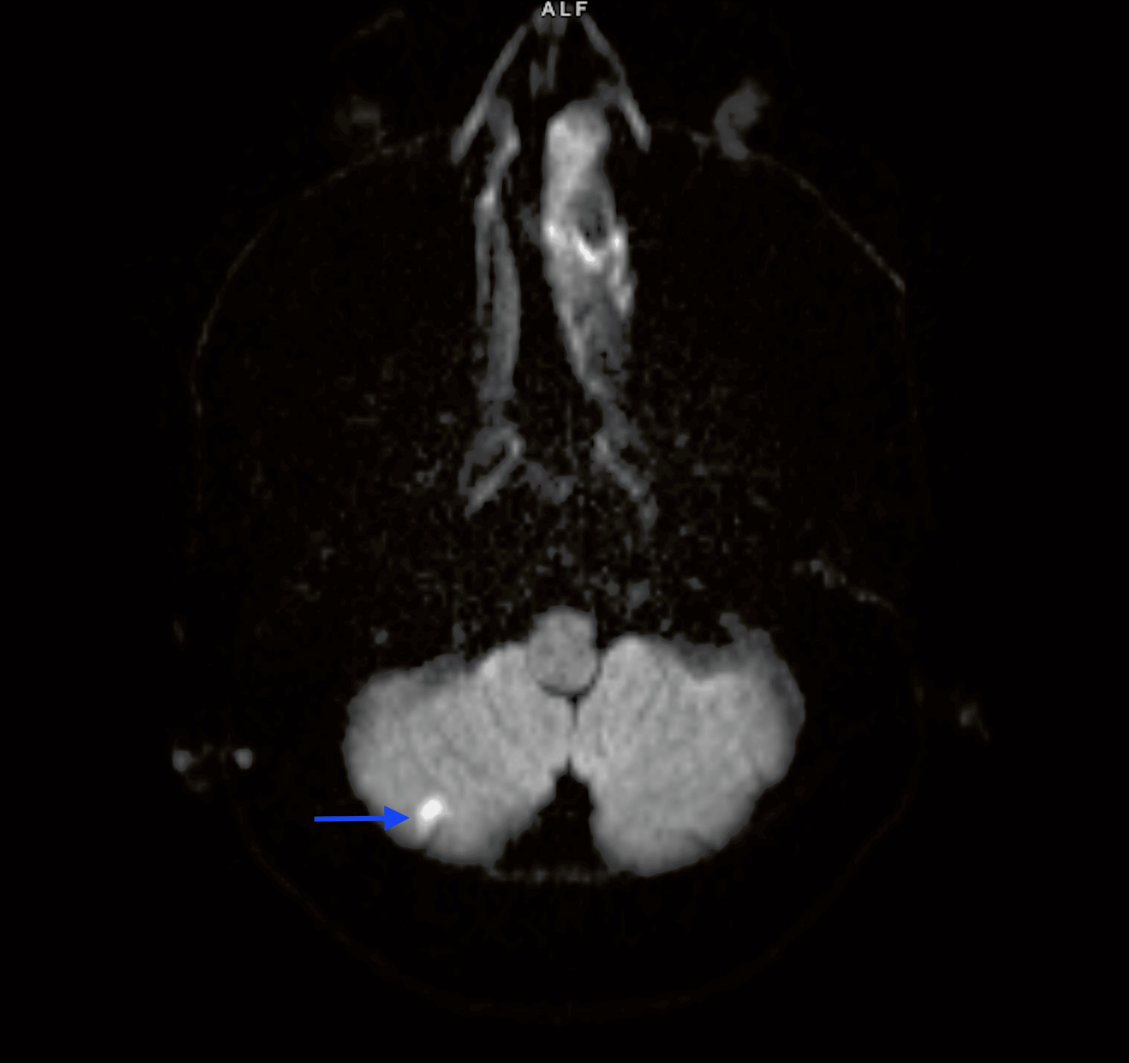
Diffusion-weighted imaging of the brain showing an area (pointed with arrow) of diffusion restriction in the right cerebellum, indicating an acute infarct.

Intravenous tPA was not offered since INR was greater than 1.7. Thrombectomy was not considered as the patient's symptoms had started to resolve and the risk of bleeding was high due to the patient’s INR level and site of the clot. A magnetic resonance imaging of the brain subsequently showed a small area of acute infarction in the right hippocampus and a punctate area of acute lacunar infarction within the right cerebellar hemisphere (Figure 3).

Since the second stroke occurred while the patient's INR was in an elevated INR-goal range of 2-2.5, the workup was extended to rule out other causes of embolic stroke. Cinefluoroscopy confirmed adequate morphology and mobility of the prosthetic AV leaflets with no turbulence of blood passing through the valve. A transesophageal echocardiogram showed a well-functioning mechanical aortic valve without restricted leaflet motion or evidence of thickening or thrombus formation. It also revealed a patent foramen ovale measuring 0.62 cm in length and 0.24 cm in height, with evidence of a left-to-right shunt. There were no thrombi or masses visualized in the aorta, left atrium, or left atrial appendage, and no residual flow was observed across the patched VSD. Mitral valve leaflets showed normal morphology and full mobility. The workup for antiphospholipid syndrome and factor V Leiden deficiency was normal. Cholesterol levels were 175 mg/dL, and low-density lipoprotein (LDL) was 118 mg/dL. A 24-hour telemetry monitoring during the patient's four-day hospital stay showed no arrhythmias, while outpatient telemetry is scheduled for next month.

To prevent future episodes of embolic stroke, the patient’s INR goal was increased to 2.5-3.5. Oral aspirin 81 mg once daily was restarted, and oral atorvastatin 40 mg once daily was added to the medical regimen, considering his LDL and cholesterol levels.

Symptoms improved and the patient was discharged with a plan for a cardiac event monitor and repeat transthoracic echocardiogram at 3 months to rule out paroxysmal atrial fibrillation and valvular thrombus, respectively. Out-patient hematology consultation was arranged to rule out a hypercoagulable state, though less likely due to the absence of a history of clot formation before the AV replacement. 

On the 6-week follow-up, the patient reported mild nighttime headaches but no nausea, vomiting, photophobia, or visual symptoms and denied any residual neurological symptoms from the stroke. The size of the clot and hence the vessel involved was smaller with a higher INR (second stroke as compared to the first). Hence, by elevating the INR goal to 2.5 to 3.5 and addition of Aspirin, we expect to prevent further ischemic strokes. If cardiac embolism continues despite elevated INR goals, the valve might need to be replaced with a bovine or the patient’s pulmonary valve.

Patent Foramen ovale-related stroke was not on the differential due to absent signs and symptoms of any peripheral thrombus during either stroke episode, hence Doppler ultrasound of legs was not done.

## Discussion

The development of recurrent thromboembolic stroke in patients with an On-X AV, despite maintaining an INR range higher than the AHA-ACC recommended range and the absence of other correctable pro-coagulant factors, presents a dilemma for preventing further stroke episodes. Risk factors for thromboembolism in patients with a mechanical heart valve, including misaligned valve leaflets and turbulent blood flow through the valve, can be checked via Cinefluoroscopy. Current literature on the utility of surveillance transthoracic or transesophageal echocardiography is unclear. Additionally, the value of echocardiography in stroke management is uncertain, as studies show that most patients with stroke or transient ischemic attack have a normal transthoracic echocardiogram, with only a few having clinically actionable findings for secondary stroke prevention [4]. Other potential causes of cardiac embolism after AV replacement include post-procedural atrial fibrillation. As per the New York State inpatient database of 122,765 patients, the incidence of atrial fibrillation can be as high as 50.1 % [5]. Therefore, in addition to a thorough evaluation of the mechanical heart valve, long-term rhythm monitoring is necessary for patients who experience embolic events following valve replacement. Current stroke guidelines recommend prolonged rhythm monitoring is suitable for the detection of atrial fibrillation within six months of cryptogenic stroke. Also, implantable cardiac monitors are becoming increasingly valuable in lowering the risk of future strokes [6].

Although transesophageal echocardiogram is the gold standard to rule out left atrial appendage thrombus, cardiac CT with delayed imaging is emerging as a reliable non-invasive alternative without compromising the diagnostic accuracy of the test [7]. This tool can be considered for clot surveillance in certain high-risk patient populations. Additionally, contrast-enhanced multi-detector cardiac CT scans are a valuable adjunct to transthoracic and transesophageal echocardiography for the precise evaluation of prosthetic valve function [8,9]. 4D imaging can assess leaflet mobility, valve stability, and the measurement of opening and closing angles, as well as the integrity of the sewing ring.

Regarding anticoagulation, current AHA-ACC recommendations suggest that, even for patients without thromboembolic risk factors, a low INR range of 1.5-2 can be effective if the patient is on both warfarin and aspirin, starting three months after On-X AV replacement (Class 2b) [3]. The absence of aspirin in our patient at the onset of both ischemic events could have been a precipitating factor for the development of thromboembolism.

Expressive aphasia typically occurs with injury to Broca’s area in the dominant frontal lobe. Crossed Aphasia is a rare variant where language deficit develops with lesions in the nondominant hemisphere [10]. The first stroke episode in this case was a crossed aphasia, where our right-handed patient, with no personal or family history of left-handedness, developed Broca's aphasia due to occlusion of the right middle cerebral artery. Additionally, the patient exhibits a co-dominant coronary blood supply, which could suggest an association between rare anatomic patterns.

## Conclusions

The occurrence of embolic strokes in a patient with On-X AV, despite adequately elevated INR levels and no correctable pro-coagulant factors, poses a significant clinical dilemma and prevention can be challenging. Current options include increasing the INR range, preventing clot formation by inhibiting multiple steps (e.g., adding aspirin), and using statins if applicable.

This patient also had an atypical presentation of stroke. It manifested as crossed aphasia, i.e., a right-handed patient developing Broca’s aphasia in case of a right-sided brain stroke. This rare anomaly supports the dissociation between handedness and linguistic hemispheric dominance.

Recommending an INR range of 1.5-2 with an On-X aortic valve requires caution. Aspirin should be strongly considered alongside warfarin, regardless of the presence of thromboembolic risk factors. Patients who develop thromboembolism have a higher risk of recurrence; therefore, a higher INR, along with aspirin and statin therapy, should be considered. Studies are needed to establish guidelines for a reliable INR range in such scenarios. Based on this case, an INR higher than 2.5 may be more appropriate.

The concomitant presence of crossed aphasia and co-dominant coronary blood supply could point toward a hypothesis and potential to study if individuals with atypical coronary anatomy likely exhibit uncommon neural function patterns.
